# Percutaneous sequential closure of an Abernethy malformation: A case report

**DOI:** 10.1016/j.radcr.2023.06.019

**Published:** 2023-06-25

**Authors:** Asad Malik, Muhammed Ebrahim Patel, Daniel Ganger, Elias Hohlastos, Ahsun Riaz

**Affiliations:** a Division of Interventional Radiology, Department of Radiology, Northwestern University, 676 N. St. Clair, Suite 800, Chicago, IL, USA; b Department of Medicine, Section of Gastroenterology/Hepatology, Northwestern University, Chicago, IL, USA

**Keywords:** Abernethy malformation, Congenital extrahepatic portosystemic shunt, Endovascular stenting

## Abstract

Abernethy malformation (congenital extrahepatic portosystemic shunt [CEPS]) is rare and is characterized by an aberrant connection between the portal and systemic veins, bypassing the liver. It can have varying presentations and can lead to severe complications if left untreated. It is usually diagnosed incidentally on abdominal imaging. Occlusion venography and measurement of portal pressures (pre- and postocclusion) is an important step in management. Complete occlusion of the malformation in cases where the portal veins in the liver are very small and the gradient is more than 10 mm Hg, can potentially lead to acute portal hypertensive complications, such as porto-mesenteric thrombosis. We report a case of Abernethy malformation diagnosed on an abdominal computed tomography scan that presented with neurological symptoms and was successfully managed by interventional radiology via endovascular closure through placement and sequential occlusion of 2 metal stents.

## Introduction

Abernethy malformation (congenital extrahepatic portosystemic shunt [CEPS]) is a rare disease with an estimated prevalence of 1 case per 30,000-50,000 births [1]. It is an aberrant connection between the mesenteric and systemic veins, caused by the abnormal involution of umbilical and vitelline veins during the embryonic period [2]. This anomaly leads to the shunting of venous blood directly from mesenteric to systemic veins, bypassing the liver and portal vein (PV) [3,4].

CEPS can be classified into 2 types; type 1 (end-to-side shunt between extrahepatic portal vein [EPV] and inferior vena cava [IVC], intrahepatic veins are absent with resulting complete absence of intrahepatic flow) and type 2 (side-to-side shunt between EPV and IVC, intrahepatic veins are present [but hypoplastic], some intrahepatic flow is present) [5].

Abernethy malformation can have varying presentations, from asymptomatic to severe manifestations with high morbidity. Some of the findings and complications are as follows: a) abnormal labs (eg, hyperammonemia, hypergalactosemia, abnormal liver function tests); b) hepatic encephalopathy and neurologic dysfunction; c) liver atrophy and failure (due to decreased hepatic blood flow); d) pulmonary hypertension; e) hepatopulmonary syndrome; and f) increased risk of hepatic neoplasia (eg, focal nodular hyperplasia, hepatic adenoma, hepatocellular carcinoma) [4], [5], [6].

CEPS is often first diagnosed in both symptomatic and asymptomatic (as an incidental finding) patients using ultrasound (US). Absence of PVs can be observed on US. However, subtle extrahepatic shunts can be difficult to be appreciated on US and therefore, Doppler US, computed tomography (CT), magnetic resonance imaging (MRI) or CT/ MRI angiography studies are employed to confirm the diagnosis and define the anatomy [7]. These modalities can help specifically in delineating the hepatic vessels, identifying the size, course, and location of extrahepatic shunts, and revealing the commonly associated congenital malformations [4,7].

We hereby report a case of Abernethy malformation that was successfully managed by interventional radiology (IR) via endovascular closure through stenting.

## Case report

A 21-year-old African American male was referred to IR with the primary complaint of increasing forgetfulness and inattentiveness that had impacted the ability to attend school. Other pertinent history includes gastroesophageal reflux disease, attention deficit hyperactivity disorder, disruptive mood dysregulation disorder and learning disabilities.

**Fig. 1:**
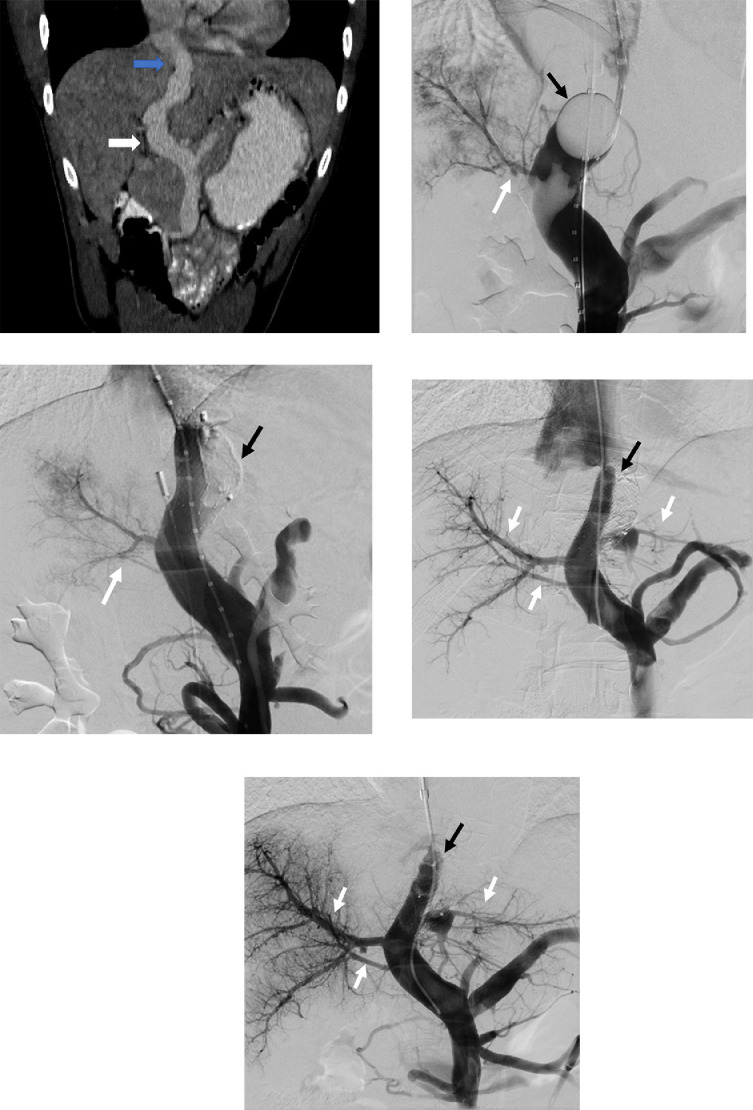
A 21-year-old male with a congenital extrahepatic portosystemic shunt (Abernethy malformation). (A) Findings: Coronal CT image obtained prior to the intervention demonstrates a large portosystemic shunt with the portal vein (white arrow) draining into the inferior vena cava via the middle hepatic vein (blue arrow), just before the cavo-atrial junction; (B) Balloon (black arrow) occlusion venogram before the first procedure shows a diminutive portal vein (white arrow); (C) Venogram following occlusion of one of the 2 parallel stents (black arrow) with slight improvement in portal vein size; (D) Venogram before the second procedure (performed 2 months after the first procedure) shows relatively developed intrahepatic portal veins (white arrows) with continued filling of the malformation via the patent stent (black arrow); (E) Venogram following occlusion of the second stent (black arrow) with significant improvement in portal vein filling (white arrows).

The patient was first diagnosed with an Abernethy malformation CEPS at the age of 20 years, when they presented with gastrointestinal symptoms (upper abdominal pain, nausea, vomiting) and elevated serum ammonia levels (96 µmol/L) which prompted an abdominal computed tomography (CT) scan. The CT scan demonstrated a portosystemic shunt (with the PV draining into the IVC via the middle hepatic vein (HV), just below the entrance of the IVC into the right atrium [RA]) and cardiomegaly (Fig. 1A). The patient was then discharged with lactulose and rifaximin and advised to await a multidisciplinary meeting between surgeons, gastroenterologists, and interventional radiologists.

Laboratory results showed a serum total bilirubin of 1.1 mg/dL (normal: 0.0-1.0 mg/dL), serum potassium of 3.4 mEq/L (normal: 3.5-5.1 mEq/L) and prothrombin time of 14.4 seconds (normal: 10.5-13.5 seconds). All other laboratory markers were within normal range.

The multidisciplinary team decided to proceed with endovascular closure of the patient's shunt by IR. The venogram of the non-occluded shunt demonstrated brisk flow from the mesenteric vein to the middle HV. Before performing the therapeutic intervention, balloon occlusion was performed to assess pre- and postshunt occlusion pressure gradients, evaluate shunt morphology, and observe filling of the intrahepatic PVs. The portosystemic gradient without shunt occlusion was 3 mm Hg and the gradient with shunt occlusion was 22 mm Hg. The minimum shunt diameter was 20 mm and the length between the HV confluence and PV measured 4 cm.

**Fig. 2:**
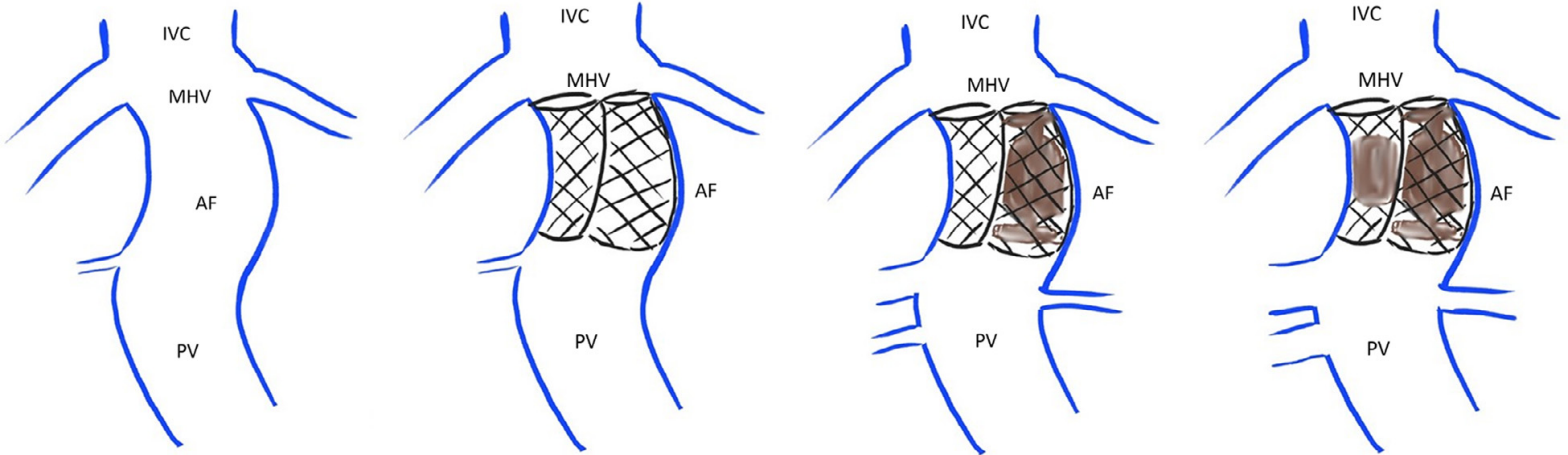
Diagrammatic representation of the treatment plan. (A) Abernethy malformation present in the patient with portal vein (PV) draining directly into the inferior vena cava (IVC) via the middle hepatic vein (MHV) through the congenital portosystemic shunt; (B and C) During the first procedure, 2 parallel stents will be placed with plug occlusion of only one of the 2 stents; (D) The second stent will be occluded during the second procedure, which will be performed 2 months after the first procedure. The portal veins would have been matured by this time.

A staged treatment was planned, as demonstrated in Fig. 2A–D. The first stage of the proposed intervention was performed under general anesthesia. The right internal jugular (IJ) and right common femoral veins were accessed, and sheaths were placed into each access site. Side by side wire access of the portal inflow of the CEPS was obtained via the neck sheath. A balloon occlusion catheter was placed at the superior aspect of the shunt and a marking pigtail catheter was placed in the shunt below the level of the balloon (Fig. 1B). The groin access was used to place an intracardiac echocardiography (ICE) catheter. Occlusion balloon venography demonstrated the location of hepatic venous malformation outflow and the location of portal venous inflow (Fig. 1B). Two Smart self-expanding stents (Cordis; Miami Lakes; FL) with a diameter of 14 mm and a length of 4 cm were deployed simultaneously side by side within the malformation; care was taken to ensure that the stents stayed above the PV and below the right HV. Self-expandable stents were selected to enable controlled deployment of the 2 parallel stents and reduce the risk of subsequent stent deformation caused by mechanical issues arising from the embolization. An Amplatzer vascular plug (Abbott; Abbott Park; IL) was then carefully deployed to occlude one of the stents (Fig. 1C). Repeat venography demonstrated complete occlusion of the medial stent, brisk flow through the lateral stent, and slightly improved portal venous flow. A portosystemic gradient of 8 mm Hg (portal 15 mm Hg, RA 7 mm Hg) was obtained after the first procedure. The total fluoroscopy time for this procedure was 32.9 minutes. The patient was discharged home without any procedure-related complications.

One month after the first intervention, the patient presented to the emergency department with abdominal pain, vomiting and weight loss. Complete blood count and LFTs were within normal limits. An abdomen/pelvis CT scan was performed, which showed periportal edema (without thrombosis) and appropriate positioning of the stents and the plug. These symptoms (pain and vomiting) improved with conservative management and the patient was sent home.

As scheduled, the patient presented to IR for stage 2 of the treatment which included occlusion of the second stent, 2 months following closure of the first stent. An 8 F x 35 cm sheath was advanced into the IVC from the right IJ access. Using a multipurpose catheter and a J glide wire (wire with a flexible tip), the lateral stent was accessed, while carefully avoiding engagement of the stent interstices. Portal venogram was performed, demonstrating significantly improved intrahepatic portal venous flow and presence of 2 additional PV branches (Fig. 1D). There was continued filling of the malformation via the patent stent. Side-by-side wire access was obtained through the nonoccluded stent. Pre- and postballoon occlusion pressure measurements did not demonstrate a significant change in the porto-systemic gradient. An Amplatzer vascular plug (Abbott; Abbott Park; IL) was deployed within the nonoccluded stent. Completion venography demonstrated complete occlusion of both stents, with improved flow through the well-developed intrahepatic PVs (Fig. 1E). The final portal pressure was 14 mm Hg, RA pressure was 11 mm Hg and portosystemic gradient was 3 mm Hg. Total fluoroscopy time for the second procedure was 9.5 minutes. The patient was then transferred to postanesthesia care unit for recovery, and eventually discharged home without complications.

There were no hospital visits or readmissions after that. The serum ammonia levels before and after the therapeutic intervention were 96 µmol/L and 33 µmol/L, respectively. The patient was monitored for 4 months following the procedure, during which there were no hospital visits or readmissions. Subsequently, the patient's care was transferred to the Hepatology team for long-term follow-up.

## Discussion

This report has demonstrated that when the pressure gradient between the preocclusion and postocclusion venography is more than 10 mm Hg, staged endovascular closure can be successfully performed by IR.

Occlusion venography for the measurement of portal pressure (eg, with balloon occlusion of the shunt) is an important diagnostic technique. It helps evaluate the anatomy and simulate the physiology of a shunt pre and postprocedure. This information is crucial in planning the most appropriate therapeutic intervention [4,8].

CEPS can occur concomitantly with other congenital abnormalities; for instance, cardiac (eg, atrial/ventricular septal defects, tetralogy of Fallot, and patent ductus arteriosus) and hepatobiliary (eg, biliary atresia, intrahepatic gallbladder, congenital hepatic fibrosis, hepatic steatosis) anomalies. This has been attributed to either genetic mutations or a common insult resulting in damage to multiple organs during the embryonic period and or to cardiac maladaptive remodeling due to altered systemic circulation hemodynamics given the presence of a shunt [9,10].

It is also important to differentiate Abernethy malformation (CEPS) from other etiologies that can present with closely resembling signs and symptoms. Some differential diagnoses are acquired (noncongenital) portosystemic shunts and surgical portosystemic shunts [7].

The treatment options available for the management of CEPS are a) surgical closure; b) percutaneous endovascular closure; and c) liver transplantation. The surgical and percutaneous interventions can be performed in a single session or in multiple sessions (staged therapy). If there is a porto-systemic gradient of more than 10 mm Hg on balloon occlusion, single stage closure carries the risk of acute portal hypertensive complications, such as porto-mesenteric thrombosis [4,[10], [11], [12].

Selecting the most appropriate therapeutic option depends on the type of malformation, presence/absence of intrahepatic veins, signs/symptoms, complications, and associated abnormalities. Typically, type 1 malformation is managed by liver transplantation (as the intrahepatic flow is completely absent), and type 2 malformation is managed by surgical or percutaneous closure of the shunt [10,11]. Some other indications for liver transplantation include the presence of tumor (eg, hepatocellular carcinoma) and associated biliary atresia [12].

Closure of the shunt is very efficacious in the prevention of long-term complications. It should also be considered as prophylactic therapy to prevent disease progression. Thereafter, long-term anticoagulation has also been recommended by some studies to decrease the risk of thrombosis [5].

Management of asymptomatic patients usually depends on the shunt ratio (volume of shunt flow/volume of total portal flow), which can be calculated via Doppler US. Treatment is recommended when the shunt ratio is >60% due to an increased risk of hepatic encephalopathy [7]. Following shunt closure, close monitoring and long-term follow-up with blood tests and imaging are required to evaluate the vascular anatomy, blood flow, functional status of the liver and to monitor for complications [4].

## Conclusion

Abernethy malformation CEPS is rare and can lead to complications if left untreated. This case report demonstrates that staged percutaneous closure can be performed by IR if the pressure gradient between pre- and postballoon occlusion venography is greater than 10 mm Hg.

## Patient consent

Written informed consent has been obtained from the patient.
